# Higher risk of colorectal cancer in patients with newly diagnosed diabetes mellitus before the age of colorectal cancer screening initiation

**DOI:** 10.1038/srep46527

**Published:** 2017-04-24

**Authors:** Sander de Kort, Ad A. M. Masclee, Silvia Sanduleanu, Matty P. Weijenberg, Myrthe P. P. van Herk-Sukel, Nico J. J. Oldenhof, Joop P. W. van den Bergh, Harm R. Haak, Maryska L. Janssen-Heijnen

**Affiliations:** 1 Dept. of Gastroenterology and Hepatology, Maastricht University Medical Center, Maastricht, The Netherlands; 2 GROW - School for Oncology and Developmental Biology, Maastricht, The Netherlands; 3 NUTRIM - School for Nutrition, Toxicology and Metabolism, Maastricht, The Netherlands; 4 Dept. of Epidemiology, Maastricht University Medical Center, GROW School for Oncology and Developmental Biology, Maastricht, The Netherlands; 5 PHARMO Institute for Drug Outcomes Research, Utrecht, The Netherlands; 6 Dept. of Hospital Pharmacy, VieCuri Medical Centre, Venlo, The Netherlands; 7 Dept. of Internal Medicine, VieCuri Medical Centre, Venlo, The Netherlands; 8 Dept. of Internal Medicine, Maastricht University Medical Centre, Maastricht, The Netherlands; 9 Dept. of Internal Medicine, Máxima Medical Centre, Eindhoven/Veldhoven, The Netherlands; 10 CAPHRI - School for Public Health and Primary Care, Maastricht, The Netherlands; 11 Dept. of Clinical Epidemiology, VieCuri Medical Centre, Venlo, The Netherlands

## Abstract

Type 2 diabetes mellitus (T2DM) is associated with greater risk for colorectal cancer (CRC). The age of onset of T2DM is decreasing worldwide. An increased CRC risk in young T2DM patients could be relevant for the age at which to initiate CRC screening. We report on CRC risk in T2DM patients with attention to age of diagnosis. We used pharmacy data (from 1998 to 2010) from the PHARMO Database Network linked to the Eindhoven Cancer Registry. Multivariable time-dependent Cox regression analyses were conducted to calculate hazard ratios (HR) for developing CRC comparing T2DM with non-T2DM. During 2,599,925 years of follow-up, 394 CRC cases among 41,716 diabetes patients (mean age 64.0 yr, 48% men) and 1,939 CRC cases among 325,054 non-diabetic patients (mean age 51.2 yr, 46% men) were identified. Diabetes was associated with an increased CRC risk in both men and women (HR 1.3, 95% CI 1.2–1.5), particularly in the first 6 months after T2DM diagnosis and pronounced in the proximal colon. This risk was even higher in men younger than 55 years (HR 2.0, 95% CI 1.0–3.8). T2DM was associated with a time-varying and subsite-specific increased CRC risk, which was even higher in men aged <55 years.

Patients with type 2 diabetes mellitus (T2DM) have a moderately increased colorectal cancer (CRC) risk (20–40%) and a worse prognosis after CRC diagnosis than non-diabetic persons^1^. The incidence rates of T2DM increased over the past decades, partly because of a shift to younger age at diagnosis^2^. With approximately 415 million patients with T2DM and more than 1 million CRC cases worldwide the moderately increased CRC risk in T2DM patients has now become an issue of concern^3^,^4^. The underlying mechanisms explaining this association have yet to be elucidated. It has been suggested that metabolic, hormonal and inflammatory changes associated with T2DM play a role^5^,^6^. For example, chronic hyperinsulinemia in T2DM patients leads to increased insulin-like growth factor (IGF) levels, which, in turn, accelerate the progression from adenoma to cancer^7^. The mechanisms involved are likely complex, with some factors having a protective effect (e.g. weight loss, use of biguanides, aspirin^8^,^9^) and others a harmful effect (e.g. sulfonylureas^10^). Currently, the information about CRC risk in diabetic patients, including age- and sex-specific variation, tumour location and the relation with the antidiabetic medication is sparse and controversial^1^,^11^,^12^,^13^. In view of the increasing number of initiatives for CRC screening worldwide, it is important to clarify the magnitude of CRC risk in diabetic patients. In particular patients newly diagnosed with T2DM at a younger age may benefit from earlier initiation of screening. In the Netherlands, the recently initiated (January 2014) nationwide CRC screening program starts at the age of 55 years. We therefore examined the sex-specific association between T2DM and CRC, both diagnosed before and after 55 years of age with focus on the diabetes duration, type of antidiabetic medication used and the tumour location.

## Results

Our study population consisted of 41,932 newly diagnosed T2DM patients and 283,122 non-diabetic controls (Table 1). The mean follow-up duration was 4.5 (±3.2) years and 7.4 (±4.1) years respectively. Table 1 presents both the time-dependent (used for the current analyses) and non-time-dependent (for insight into our data) baseline characteristics of the study cohort. T2DM patients were on average 12.8 years older than the non-diabetic persons (64.0 (±12.8) and 51.2 (±15.0) years respectively) (P < 0.001). Of all T2DM patients, 9,413 (23%) were younger than 55 years at the first dispense of antidiabetic medication. Of all non-diabetic controls, 201,078 (62%) were younger than 55 years at the start of the study (P < 0.001). The majority of T2DM patients used insulin sensitizers (82%), followed by insulin secretagogues (55%) whether or not in combination with insulin analogues (21%). Patients with T2DM more often used statins (69% vs. 16%, p < 0.001) and aspirin (33% vs. 11%, p < 0.001) than the non-diabetic controls.

In the 41,716 patients who developed T2DM, 394 patients were diagnosed with CRC during 189,568 person-years of follow-up. Likewise, in the non-diabetic group, 1,939 patients were diagnosed with CRC during 2,410,357 person-years of follow-up. Of these 1,939 CRC cases, 216 were diagnosed with T2DM after the CRC diagnosis (date of CRC diagnosis < date of first dispense of any drug used in diabetes) and were therefore classified as CRC in the non-diabetic group. With regard to the colonic subsite, patients with T2DM more often had proximal CRC (45% vs. 33%) and less often rectal cancer (19% vs. 29%) than non-diabetic controls (overall p < 0.001). In both T2DM and non-diabetic groups, 30% of the CRCs were distally located.

Table 2 shows the relative risk estimates for CRC, further stratified by sex- and subsite. After adjustment for age and statin use, T2DM was significantly associated with a higher risk of CRC in both men (HR 1.3, 95% CI: 1.1–1.5) and women (HR 1.3, 95% CI: 1.1–1.6). The association was greater for proximal CRC (men: HR 1.6, 95% CI 1.2–2.1; women: HR 1.8, 95% CI: 1.4–2.3), the effect decreasing in size as the cancer localization shifted from proximal to distal. T2DM was not significantly associated with distal colon and rectal cancer, neither in men nor in women. In sensitivity analyses, neither the exclusion of patients who used insulin only during follow-up (n = 2,506) nor the inclusion of prevalent T2DM patients (n = 18,857) altered the found estimates significantly (data not shown).

Table 3 shows the relative risk estimates for patients younger than 55 years at T2DM and CRC diagnosis compared to the total group. We found a stronger association between T2DM and CRC in men younger than 55 years (HR 2.0, 95% CI: 1.0–3.8 compared to HR 1.3, 95% CI: 1.1–1.5 among men in the total group), but not in women (where only 4 CRC cases were diagnosed in the younger group; HR 1.2, 95% CI 0.4–3.6 compared to HR 1.3, 95% CI: 1.1–1.6 among women in the total group).

No difference in the effect size of CRC risk was found in T2DM patients when stratifying by type of firstly dispensed antidiabetic medication. As shown in Table 4, the use of an insulin sensitizer (HR 1.3, 95% CI: 1.2–1.6) or an insulin secretagogue (HR 1.3, 95% CI: 1.1–1.5) as first antidiabetic medication dispensed was associated with a similar CRC risk. In total 2,003 of the 14,372 insulin secretagogue users and 25,765 insulin sensitizer users received dual (sensitizer and secretagogue) therapy at the time of T2DM diagnosis.

As shown in Table 5, in the first 3 months (HR 3.1, 95% CI: 1.8–5.2) as well as in the subsequent 3 months (HR 2.1, 95% CI: 1.1–3.9) after T2DM diagnosis, the risk of CRC in T2DM patients was significantly higher than in non-diabetic controls. No further increase of CRC risk was found in the following 2 three-month intervals. During the period from 6 months until the end of follow-up, the CRC risk remained increased in patients with T2DM (HR 1.3, 95% CI: 1.2–1.5), though the effect size was slightly attenuated compared to analysis in which the first 6 months of follow-up were included (HR 1.4, 95% CI: 1.2–1.6). Time split analyses performed at median half time (3.2 years in T2DM) showed similar effect sizes in the first (HR 1.3, 95% CI: 1.0–1.6) and second (HR 1.4, 95% CI: 1.2–1.6) part of the follow-up time.

## Discussion

Our study supports the role of newly diagnosed T2DM as an established risk factor for CRC. With an increased overall CRC relative risk of 30% and an increased proximal colon cancer risk of 70% found in T2DM patients compared to individuals without diabetes, our study confirms results from previous cohort studies^11^,^12^,^14^,^15^,^16^,^17^. We examined the role of T2DM as a risk factor for CRC before the age of 55 years at which in most countries population-based CRC screening starts. We found that T2DM in young patients was associated with a more pronounced increased risk of CRC diagnosis before the age of 55 years compared to the total population. After adjusting for age and statin use, we observed an increased risk for CRC diagnosis before age 55 in men with T2DM compared to non-diabetic men. In women with T2DM this risk appeared increased though statistical significance was not reached due to small numbers.

The mechanisms that underlie the association between T2DM and CRC are yet to be explored. A shared risk profile including lifestyle factors such as obesity, unfavourable diets, and low levels of physical activity that lead to metabolic abnormalities and cell proliferation (e.g. hyperinsulinemia) can partly explain the association. Hyperinsulinemia as a cancer promoting metabolic factor in T2DM patients suggests a CRC risk modifying role for anti-diabetes drugs as insulin analogues and secretagogues elevate serum insulin serum levels in contrast to insulin sensitizers that lower insulin levels. Insulin secretagogues and analogues have been associated with increased CRC risk^18^ and insulin sensitizers with a decreased CRC risk^9^,^13^,^19^. However, most of the findings from conducted pharmacoepidemiological studies are possibly methodologically biased. An example is immortality bias. Immortality time refers to a period of follow-up in which death or an outcome (CRC) cannot occur due to design of the study. For instance, waiting and surviving until a first anti-diabetic prescription (e.g. metformin) is dispensed while patient is already followed-up and classified as metformin user from cohort entry. Results are biased in favour of the treatment group due to a partial survival advantage to the non-treated group^20^. In our analyses we accounted for these potential biases as much as possible and found no difference in CRC risk or effect size when stratifying according to the first type of ADD dispensed. When interpreting these findings it should be noted that ADDs could be added, stopped or switched during follow-up. Therefore, the reported increased CRC risks in our study cannot be attributed to one type of anti-diabetes drug only.

A study conducted by Johnson *et al*.^21^ addressed the involvement of detection bias in the increased risk of CRC and other malignancies in T2DM patients. They compared T2DM with non-diabetic individuals and found an overall adjusted CRC HR of 1.2, with an initial peak in CRC risk (HR 2.8) in the first three months after diagnosis of T2DM. Exclusion of the first three months of the 10 year follow-up time resulted in a lower overall risk (HR 1.1), suggesting overestimation of the overall CRC risk estimates in T2DM patients in cohort studies. Our study confirms the initial spike in CRC risk in the first (HR 3.1) and second (HR 2.1) three months after T2DM diagnosis and a subsequent lowering of effect size of the overall CRC risk after exclusion of the first 6 months of follow-up after T2DM diagnosis. Nevertheless, T2DM remained associated with an increased CRC risk after accounting for detection bias.

Our stratified analyses showed that a T2DM diagnosis was associated with an increased risk for CRC diagnosis before the age of 55 years in men. In women no statistically significant increased risk was observed (probably due to small numbers). To our knowledge, there are no previous studies that report on this age-specific association. Although the number of CRCs diagnosed before the age of 55 years in patients with T2DM was small in our study (13 in men and only 4 in women), our data suggest that lowering the age limit of screening initiation in patients with T2DM might be beneficial. Future studies are required to investigate the cost-effectiveness of lowering the age limit of initiation of CRC screening.

The strength in our study lies in the use of population-based outpatient pharmacy data and nearly complete and detailed cancer registration data. A very large cohort could be composed in which incident T2DM patients were selected based on anti-diabetes drug use. Additionally, a high number of histologically confirmed CRCs were identified. The methodology of this study allowed us to avoid potential biases known to occur in pharmacoepidemiological studies. However, there are also several limitations to our study. Misclassification of T2DM could have occurred as some patients remained undetected, or were diagnosed with T2DM without anti-diabetic drugs being dispensed, which entails about 10% of the total diabetes population^22^. Also no clinical laboratory data (e.g. glutamic acid decarboxylase) were available to differentiate between T1DM and T2DM. Misclassification could have resulted in slight attenuation of estimated HR.

Information regarding “over the counter” drug and supplement use (vitamin D, calcium and aspirin) was not available, resulting in underestimation of the prevalence of co-medication use. Also, lack of information regarding important confounders such as body mass index, dietary habits, smoking and physical activity could have influenced our results. On the other hand, previous prospective cohort studies showed that correction for these factors only marginally attenuated relative risks. Additionally, by adjusting for concomitant statin use we aimed to partially adjust for these confounders by proxy.

In conclusion, newly diagnosed T2DM was associated with a time-varying and subsite-specific increased CRC risk, but also with an even more pronounced increased risk of CRC diagnosis before the age of initiation of CRC screening (55 years) in men. This pronounced increased risk should be reconfirmed in future studies with more confounding information, particularly on family history. The clinical importance of such increased risk and the potential benefits and cost-effectiveness of tailoring screening strategies (e.g. lowering the age limit of CRC screening) in T2DM patients need further investigation.

## Materials and Methods

Data were derived from a combined database of two Dutch research institutes (Eindhoven Cancer Registry (ECR) and the PHARMO Database Network), momentarily covering over one million individuals in the southern region of the Netherlands. Specific information on the linkage of both databases and opportunities for research have been described previously^23^. The ECR is a population-based registry maintained by the Netherlands Comprehensive Cancer Organization. The registry comprises data on all newly diagnosed cancer patients. It started in 1995 in the city of Eindhoven and gradually expanded the registration area until covering a region of 2.4 million inhabitants in the southern part of the Netherlands since 1988. Information is received and collected by trained personnel from six pathology departments, ten general hospitals and two radiotherapy institutes. The coverage of this cancer registration is over 95%^24^. The PHARMO Database Network is a population-based network of healthcare databases and combines data from different healthcare settings in the Netherlands since 1986. The data network covers a demographic region of more than 3 million inhabitants of various regions of the Netherlands. Information for this study is acquired from out-patient pharmacies and contains longitudinal drug dispensing records, including information on dispensing date. Linkage of ECR and PHARMO was performed for patients diagnosed between 01-01-1998 and 31-12-2010 who were living in an overlapping registration area with nearly complete coverage. Patients were followed-up until emigration from the PHARMO-ECR catchment area; end of out-patient pharmacy data collection; end of follow-up or death, whichever occurred first.

### Study population

**Figure 1:**
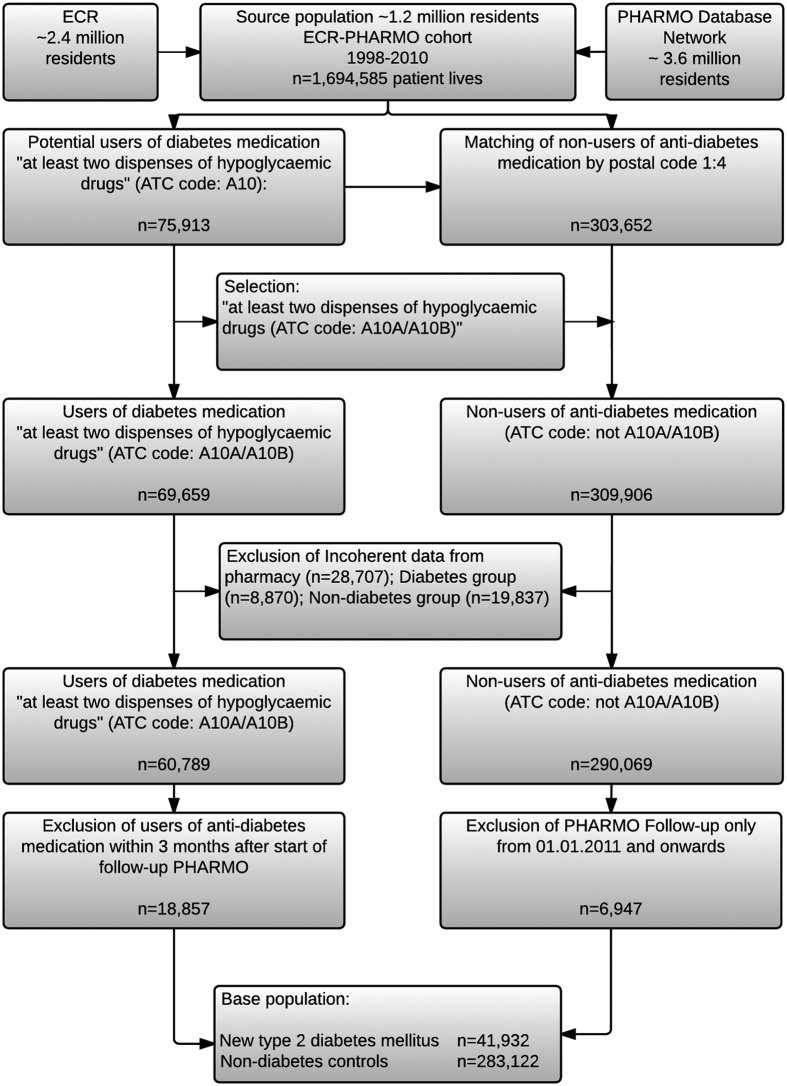
Study flowchart.

The study flow-chart is illustrated in Fig. 1. To prevent inclusion of patients with type I DM and improve comparison with previous cohort studies^14^,^25^, we included all individuals aged 30 years or older who lived in the PHARMO-ECR catchment area between 01-01-1998 and 31-12-2010. Patients with and without diabetes were identified based on coded dispensing information according to the Anatomical Therapeutic Chemical Classification System (ATC). According to a recent Swedish study by Jansson *et al*.^22^ about 90% of patients with diabetes are pharmacologically treated. Individuals registered with at least two dispenses of hypoglycaemic drug coded A10 during follow-up (insulin and analogues and blood glucose lowering drugs) were defined as potential diabetic patients (n = 75,913). Potential diabetic patients were matched only by postal code semi-randomly at a 4:1 ratio with 303,652 persons who did not receive anti-diabetes medication during the study period, to warrant equal CRC registration coverage. Of the 75,913 potential diabetic patients (ATC code containing “A10”), 69,659 had used anti-diabetes medication (ATC code containing “A10A” or “A10B”) during the study period and were defined as diabetic patients. The remaining 6,254 individuals were assigned to the non-diabetic group, as these patients were dispensed predominantly non-diabetes medication containing A10 in the ATC code (e.g. promethazine D04AA10). We excluded patients with incoherent out-patient pharmacy data (n = 28,707) and patients with follow-up in the PHARMO Database Network starting from 01.01.2011 and onwards (n = 6,947) as no linkage with the ECR was available at the time of this study. We defined newly diagnosed T2DM patients as patients who were dispensed antidiabetic medication for the first time after 3 months of follow-up (as recipes are commonly dispensed for ≤3 months). A total of 18,857 T2DM patients were dispensed antidiabetic medication within the first 3 months of follow-up and were excluded from analyses. The remaining 41,932 T2DM patients were qualified as newly diagnosed T2DM patients and were included in the analyses together with the 283,122 non-diabetic controls.

### Colorectal cancer and subsite

From 01-01-1998 to 31-12-2010, 2,333 new CRC cases (in approximately 2.6 million person years of follow-up) were registered and available for analyses (ICD-10 codes C18–C20. Among these, 819 were proximal CRCs (codes C18.0–C18.5), 699 distal CRCs (codes C18.6 and C18.7), and 642 rectal cancers (C20).

### Demographic features, antidiabetic medication and co-medication used

The covariates used in the analyses were derived from the PHARMO Database Network and included age at start of the study and sex. Analyses were stratified according to the type of antidiabetic drug (ADD) used: (1) *Insulin* s*ensitizers* (biguanides “A10BA”, thiazolidinediones “A10BG”), (2) *Insulin* s*ecretagogues* (sulfonylureas “A10BB”, dipeptidyl peptidase-4 inhibitors “A10BH”, glucagon-like peptide-1 agonists “A10BX04-07”), and (3) *insulin analogues* (“A10A”). The use of statin (ATC codes C10AA/C10BA/C10BX), aspirin (ATC codes N02BA01/B01AC06), calcium supplement (ATC code A12AA), and vitamin D supplement (ATC codes A11CC/A11CB) were also included as potential confounders, as these medications may attenuate the CRC risk^8^,^26^.

### Statistical analyses

Adjusted hazard ratios (HR) for CRC incidence were calculated using Cox regression with T2DM as a time-dependent variable entered from the date of first dispense of any ADD. This means that the person years between the ECR-PHARMO index date and the date of first ADD dispense were added to the control group. Each of the controls was assigned their own cohort entry date of first entry in the PHARMO database. Confounders were selected a priori on the basis of literature and checked for their potential to change HR by more than 10% using a backward procedure. Age and the use of statin changed the HR by more than 10% and were included in the multivariable-adjusted model. We also included sex distribution in the multivariate model, as male gender is an established risk indicator for CRC^27^. Statin use (at least 1 dispense of statin) was entered as a dichotomous (yes/no) time-dependent variable. Analyses were also stratified by gender and subsite to assess the impact of effect modification and to compare results with previous literature.

For the age-specific analyses a cut-off of 55 years of age was used, which is the starting age for initiation of CRC screening in our nationwide program. This resulted in a “young” group of T2DM patients (diagnosed <55 years of age) and controls (aged <55 years at cohort entry) who were followed-up until CRC diagnosis, age of 55 years, or end of follow-up.

To mitigate the potential impact of type I DM patients in this cohort on CRC risk estimates, we performed sensitivity analyses excluding DM patients using only insulin analogues during follow-up. Second sensitivity analyses were performed using all 60,789 prevalent and newly diagnosed T2DM patients to estimate potential selection bias. To account for time-varying HR, we also performed time-split analyses in three month intervals during the first year after diagnosis of diabetes according to a previously published study by Johnson *et al*.^21^ addressing potential detection bias. In the latter analyses, follow-up time of T2DM patients prior to diagnosis of diabetes was discarded in the analyses and thus not added to the person-years of observation for non-diabetic persons.

For all analyses, the proportional hazards assumption was tested by visual inspection of the -log-log transformed hazard curves; no violation of the latter assumption was detected. Statistical significance was tested at the 0.05 level using two-sided tests. Analyses were conducted using Stata (version 12, Statacorp, College Station, TX, USA).

## Additional Information

**How to cite this article**: de Kort, S. *et al*. Higher risk of colorectal cancer in patients with newly diagnosed diabetes mellitus before the age of colorectal cancer screening initiation. *Sci. Rep.* **7**, 46527; doi: 10.1038/srep46527 (2017).

**Publisher's note:** Springer Nature remains neutral with regard to jurisdictional claims in published maps and institutional affiliations.

## Figures and Tables

**Table 1:** Baseline characteristics of diabetic and non-diabetic individuals within the ECR-PHARMO cohort.

Characteristic	Non time-dependent				*p*-value	Time-dependent*				*p*-value
	*No T2DM (n)*	*%*	*T2DM (n)*	*%*		*No T2DM (n)*	*%*	*T2DM (n)*	*%*	
**Population**	283,122	87%	41,932	13%		325,054	89%	41,716^†^	11%	
**Mean age at inclusion (yr** **±** **sd)**	50.0 ± 14.8		59.3 ± 13.2		<0.001	51.2 ± 15.0		64.0 ± 12.8		<0.001
<55 year	185,894	66%	15,184	36%		201,078	62%	9,413	23%	
55–75 year	77,428	27%	21,892	52%		99,320	30%	23,345	56%	
>75 year	19,800	7%	4,856	12%		24,656	8%	8,958	21%	
**Gender**										
Men	130,124	46%	20,303	48%		150,427	46%	20,166	48%	
Women	152,998	54%	21,629	52%	<0.001	174,627	54%	21,550	52%	<0.001
**Mean FU time (yr** **±** **sd)**	7.8 ± 4.1		9.2 ± 3.6		<0.001	7.4 ± 4.1		4.5 ± 3.2		<0.001
**Colorectal cancer distribution**	1,723		610			1,939		394^†^		
proximal colon	588	34%	231	38%		642	33%	177	45%	
distal colon	503	29%	193	32%		581	30%	118	30%	
rectum	502	29%	140	23%		566	29%	76	19%	
NOS/rectosigmoid	130	8%	46	8%	0.023	150	8%	23	6%	<0.001
**DM medication (n, % yes)**										
has used insulin	Na	8,908	21%		na	8,871	21%			
has used insulin sensitizers	Na	34,208	82%		na	34,053	82%			
has used insulin secretagogues	Na	22,89	55%	na	na	22,778	55%	na		
**Co-medication (n, % yes)**										
Statin	39,042	14%	29,13	69%	<0.001	50,837	16%	28,964	69%	<0.001
Aspirin	27,419	10%	13,811	33%	<0.001	34,604	11%	13,716	33%	<0.001
Vitamin D	2,692	1%	1,285	3%	<0.001	3,007	1%	1,264	3%	<0.001
Calcium supplement	6,076	2%	1,801	4%	<0.001	6,998	2%	1,785	4%	<0.001

T2DM type 2 diabetes mellitus; sd standard deviation; NOS not otherwise specified; FU follow-up.

*Differences in (n) between non-time-dependent and time-dependent baseline values occur when T2DM patients have FU time as NoT2DM individuals in time-dependent analyses.

^†^In 216 T2DM patients, colorectal cancer diagnosis occurred before diagnosis of T2DM.

**Table 2:** Gender- and subsite specific time-dependent cox-proportional hazard analyses on the association between type 2 diabetes mellitus and colorectal cancer.

	CRC Cases (T2DM/non-T2DM)	PY (T2DM/non-T2DM)	HR^*^ (95% CI)	HR^†^ (95% CI)
**Men and Women**				
Colorectal cancer	394/1,939	189,568/2,410,357	1.4 (1.3–1.6)	1.3 (1.2–1.5)
Proximal colon cancer	177/642	189,568/2,410,358	1.8 (1.5–2.2)	1.7 (1.4–2.0)
Distal colon cancer	118/581	189,568/2,410,359	1.4 (1.2–1.7)	1.2 (1.0–1.5)^‡^
Rectal cancer	76/566	189,568/2,410,360	1.0 (0.8–1.3)	1.0 (0.7–1.3)
**Men**				
Colorectal cancer	219/1,092	94,612/1,149,183	1.4 (1.2–1.7)	1.3 (1.1–1.5)
Proximal colon cancer	81/323	94,612/1,149,184	1.7 (1.3–2.2)	1.6 (1.2–2.1)
Distal colon cancer	74/338	94,612/1,149,185	1.5 (1.2–2.0)	1.3 (1.0–1.7)^§^
Rectal cancer	52/350	94,612/1,149,186	1.1 (0.8–1.5)	1.1 (0.8–1.5)
**Women**				
Colorectal cancer	175/847	94,956/1,261,174	1.5 (1.2–1.7)	1.3 (1.1–1.6)
Proximal colon cancer	96/319	94,956/1,261,175	2.0 (1.6–2.5)	1.8 (1.4–2.3)
Distal colon cancer	44/243	94,956/1,261,176	1.3 (0.9–1.8)	1.2 (0.8–1.7)
Rectal cancer	24/216	94,956/1,261,177	0.8 (0.6–1.3)	0.8 (0.5–1.2)

PY person-years; T2DM type 2 diabetes mellitus; CRC Colorectal cancer; HR hazard ratio.

*Adjusted for age and gender (mixed group).

^†^Adjusted for age, gender (mixed group) and statin use.

^‡§^Not statistically significant (^‡^*p* = 0.052; ^§^*p* = 0.081).

**Table 3:** Gender specific analyses on the association between type 2 diabetes mellitus and colorectal cancer diagnosed before the age of 55 years compared to the total group.

	CRC Cases (T2DM/non-T2DM)	PY (T2DM/non-T2DM)	HR^*^ (95% CI)	HR^†^ (95% CI)
**Diagnosis of T2DM and CRC <55 years**				
Men and women	17/215	37,658/1,327,066	1.8 (1.1–2.9)	1.7 (1.0–3.0)^‡^
Men	13/128	21,796/662,967	1.9 (1.1–3.4)	2.0 (1.0–3.8)
Women	4/87	15,862/674,099	1.4 (0.5–3.7)	1.2 (0.4–3.6)
**Total group**				
Men and women	394/1,939	189,568/2,410,357	1.4 (1.3–1.6)	1.3 (1.2–1.5)
Men	219/1,092	94,612/1,149,183	1.4 (1.2–1.7)	1.3 (1.1–1.5)
Women	175/847	94,956/1,261,174	1.5 (1.2–1.7)	1.3 (1.1–1.6)

T2DM diabetes mellitus; CRC colorectal cancer; PY person-years; HR hazard ratio.

*Adjusted for age and gender (mixed group); ^†^adjusted for age, gender (mixed group) and statin use, ^‡^not statistically significant (p = 0.052).

**Table 4:** Anti-diabetic medication specific analyses on the association between type 2 diabetes mellitus and colorectal cancer.

	CRC Cases (T2DM/non-T2DM)	PY (T2DM/non-T2DM)	HR* (95% CI)	HR^†^ (95% CI)
**According to 1st anti-DM prescription**				
After any 1st prescription for diabetes	394^‡^/1939	189,568/2,410,357	1.4 (1.3–1.6)	1.3 (1.2–1.5)
After 1st prescription = sensitizer	191/1939	94,237/2,410,357	1.5 (1.3–1.8)	1.3 (1.2–1.6)
After 1st prescription = secretagogue	190/1939	85,415/2,410,357	1.4 (1.2–1.6)	1.3 (1.1–1.5)
After 1st prescription = insulin analog	32/1939	20,541/2,410,357	1.3 (0.9–1.8)	1.2 (0.8–1.7)
After 1st prescription = double prescription	19/1939	10,655/2,410,357	1.3 (0.8–2.0)	1.1 (0.7–1.8)

T2DM type 2 diabetes mellitus; HR hazard ratio; PY person-years; CRC colorectal cancer.

*Adjusted for age and gender (mixed group). ^†^adjusted for age, gender (mixed group) and statin use.

^‡^Counts do not add up as 19 CRC patients with T2DM start with double anti-DM medication at diagnosis of T2DM.

**Table 5:** Type 2 diabetes mellitus and colorectal cancer risk according to different time periods after T2DM diagnosis.

	CRC Cases (T2DM/non-T2DM)	PY (T2DM/non-T2DM)	HR^*^ (95% CI)	HR^†^ (95% CI)
**Start - end of FU**	394/1,723	189,568/2,212,801	1.5 (1.4–1.7)	1.4 (1.2–1.6)
**First year of FU**				
Start - 3 months	33/38	10,277/70,420	3.0 (1.9–4.9)	3.1 (1.8–5.2)
3 Months - 6 months	20/35	9,931/69,489	2.0 (1.1–3.4)	2.1 (1.1–3.9)
6 Months - 9 months	12/40	9,593/68,486	1.1 (0.6–2.1)	0.9 (0.4–1.9)
9 Months - 12 months	15/48	9,235/67,503	1.1 (0.6–2.0)	1.3 (0.7–2.4)
**Exclusion of the first 6 months**				
6 Months - end of FU	341/1,650	169,360/2,072,892	1.5 (1.3–1.7)	1.3 (1.2–1.5)
**Split at median FU time**				
6 Months - 3.2 year	144/443	85,398/688,028	1.3 (1.1–1.6)	1.3 (1.0–1.6)
3.2 Year - end of FU	197/1,207	83,962/1,384,864	1.6 (1.4–1.9)	1.4 (1.2–1.6)

CRC colorectal cancer; T2DM type 2 diabetes mellitus; FU Follow-up; HR hazard ratio; PY person-years.

^*^Adjusted for age and gender; ^†^adjusted for age, gender and statin use.
